# Repair of symptomatic superior mesenteric artery pseudoaneurysm with arteriovenous fistula using physician-modified endograft

**DOI:** 10.1016/j.jvscit.2024.101646

**Published:** 2024-10-22

**Authors:** Olivia Fuson, Claire Janssen, Andrew Barleben, Ann Gaffey

**Affiliations:** Department of Vascular and Endovascular Surgery, University of California at San Diego, San Diego, CA

**Keywords:** Superior mesenteric artery, Pseudoaneurysm, Fistula

## Abstract

Superior mesenteric artery (SMA) pseudoaneurysm with superior mesenteric arteriovenous fistula (SMAVF) is a rare pathology associated with high rates of rupture and mortality. Known interventions for the treatment of SMA pseudoaneurysm with SMAVF include open repair or endovascular repair with coil embolization or covered stenting. To the best of our knowledge, this report is the first of physician-modified endograft for the treatment of SMA pseudoaneurysm with SMAVF after prior thrombosis, ligation, and coil embolization of the SMA. The patient recovered well and has 1 month of follow-up after the procedure.

Superior mesenteric artery (SMA) pseudoaneurysm with superior mesenteric arteriovenous fistula (SMAVF) is a rare and dangerous pathology requiring immediate intervention. Prior reported methods of treatment for SMA pseudoaneurysm with SMAVF include open repair or endovascular repair with coil embolization or covered stenting. In this particular case, we illustrate a novel technique of physician-modified endograft (PMEG) with exclusion of the SMA as a method to treat SMA pseudoaneurysm with SMAVF after prior thrombosis, ligation, and coil embolization of the SMA. The patient provided written informed consent for this publication.

## Case report

**Fig 1:**
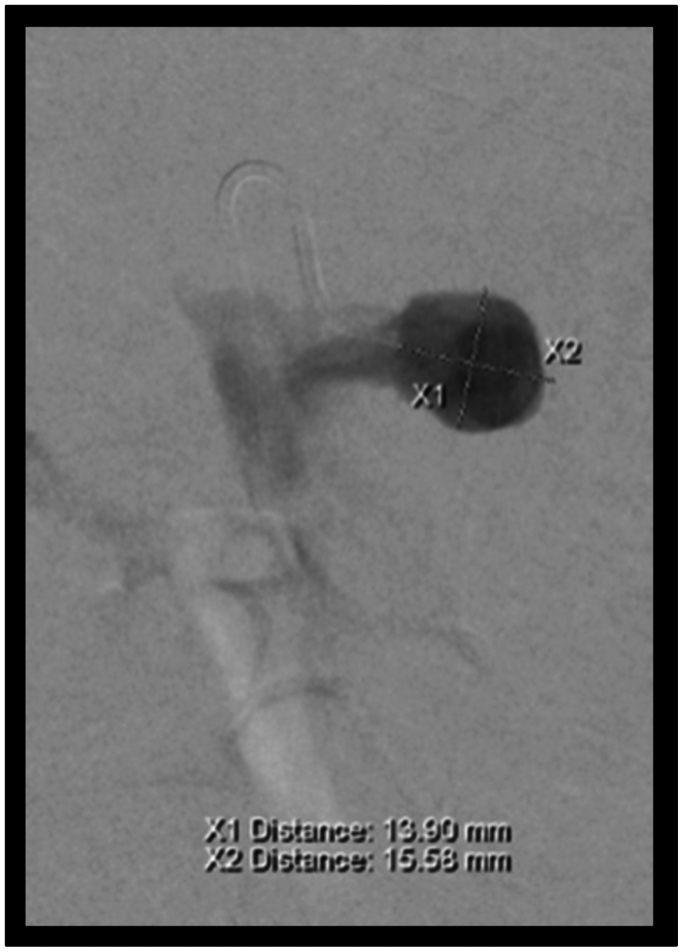
Angiography of initial pseudoaneurysm, before coiling.

A 61-year-old man with a history of pancreatic cancer previously treated with chemotherapy, radiation, and intraoperative radiotherapy with gastrojejunostomy creation presented to our hospital with severe abdominal pain. Computed tomography scan of the abdomen and pelvis revealed distal occlusion of the SMA and a proximal pseudoaneurysm (Fig 1). The patient underwent coil embolization of the SMA and was discharged home in stable condition after resolution of his abdominal pain.

Within 1 month, he represented to an outside hospital emergency department with recurrent abdominal pain. Imaging revealed acute cholecystitis and he underwent cholecystostomy tube placement. Magnetic resonance imaging revealed recurrence of SMA pseudoaneurysm. There was no evidence of external compression from the tumor. Abdominal pain resolved with percutaneous biliary drain placement and biliary stenting and the patient was discharged to home with outpatient follow-up.

**Fig 2:**
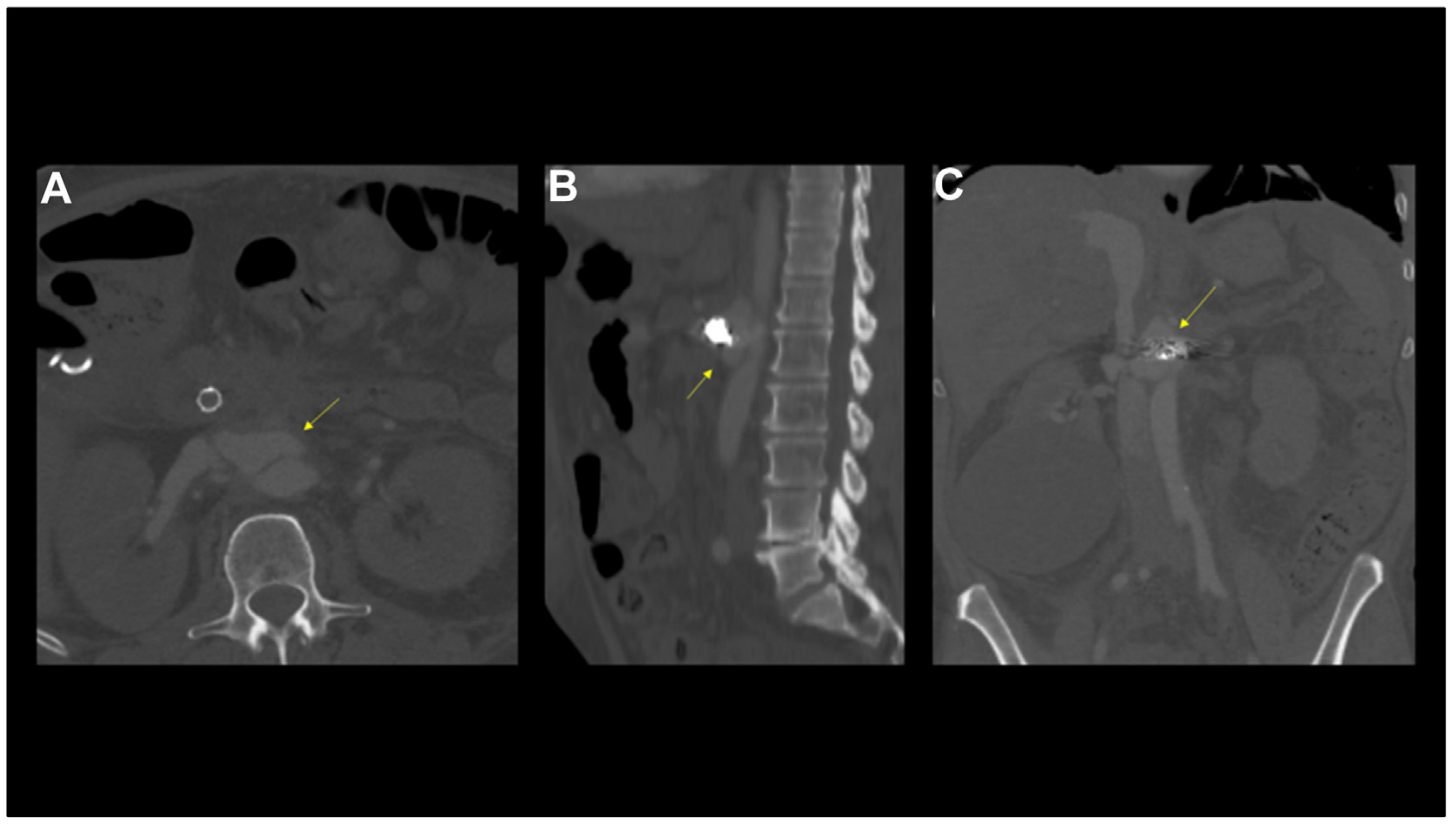
Axial **(A)** and coronal **(B)** view of the pseudoaneurysm. Sagittal **(C)** view of the arteriovenous fistula with arterial phase filling of the inferior vena cava.

One month later, he presented again with new-onset symptoms of bilateral lower extremity swelling with 3+ pitting edema along the course of his legs. Echocardiogram revealed high-output heart failure. The cholecystostomy tube was still in place. Duplex evaluation was negative for a deep venous thrombosis. A repeat CTA revealed an increased size of the SMA pseudoaneurysm (1.8 × 3.8 × 4.1 cm), as well as a newly diagnosed arteriovenous fistula between the SMA aneurysm coiled stump and adjacent left renal vein/inferior vena cava (Fig 2).

After a discussion with the oncology service and results from a positron emission tomography scan with magnetic resonance imaging, which revealed no significant change in poorly defined pancreatic head/uncinate mass and no specific evidence for new upper abdominal metastases, the patient was deemed to be amenable to surgical intervention. His last intervention for the pancreatic mass had been an endoscopic retrograde cholangiopancreatography 3 months before presentation to this hospital. Given his prior open surgeries, the decision was made to proceed with endovascular intervention to exclude the pseudoaneurysm by reducing the inflow with a PMEG. On the back table, a 26 mm × 79 mm Cook alpha graft (Cook Medical, Bloomington, IN) with fenestrations for the celiac artery, left renal, and right renal was prepared. Given the possible concern for infection, the graft was flushed with rifampin.

Under general anesthesia, bilateral percutaneous femoral access was obtained. Notably, the patient did become hypotensive with induction of anesthesia and required vasopressor support. Delayed angiogram revealed retrograde filling of SMA branches via the arc of Riolan and a meandering mesenteric artery, and the decision was made to proceed with graft placement. The graft was delivered through the right groin, unsheathed, and we confirmed the location of the celiac fenestration with the CYDAR overlay (a virtual reconstruction of the patient's aorta based on computed tomographic imaging, which guides vessel selection intraoperatively). Once the graft was unsheathed, the patient's hypotension resolved and vasopressors were discontinued. From the left femoral access, we then individually selected the celiac, left renal artery, and right renal artery with an Oscor deflectable sheath. We placed stiff wires into all of these vessels after catheter confirmation and angiogram for each vessel from the left common femoral artery. We subsequently stented the visceral arteries using Viabahn balloon-expandable (W. L. Gore & Associates, Flagstaff, AZ) stents. All stents were post-dilated. The graft was ballooned with a compliant balloon.

**Fig 3:**
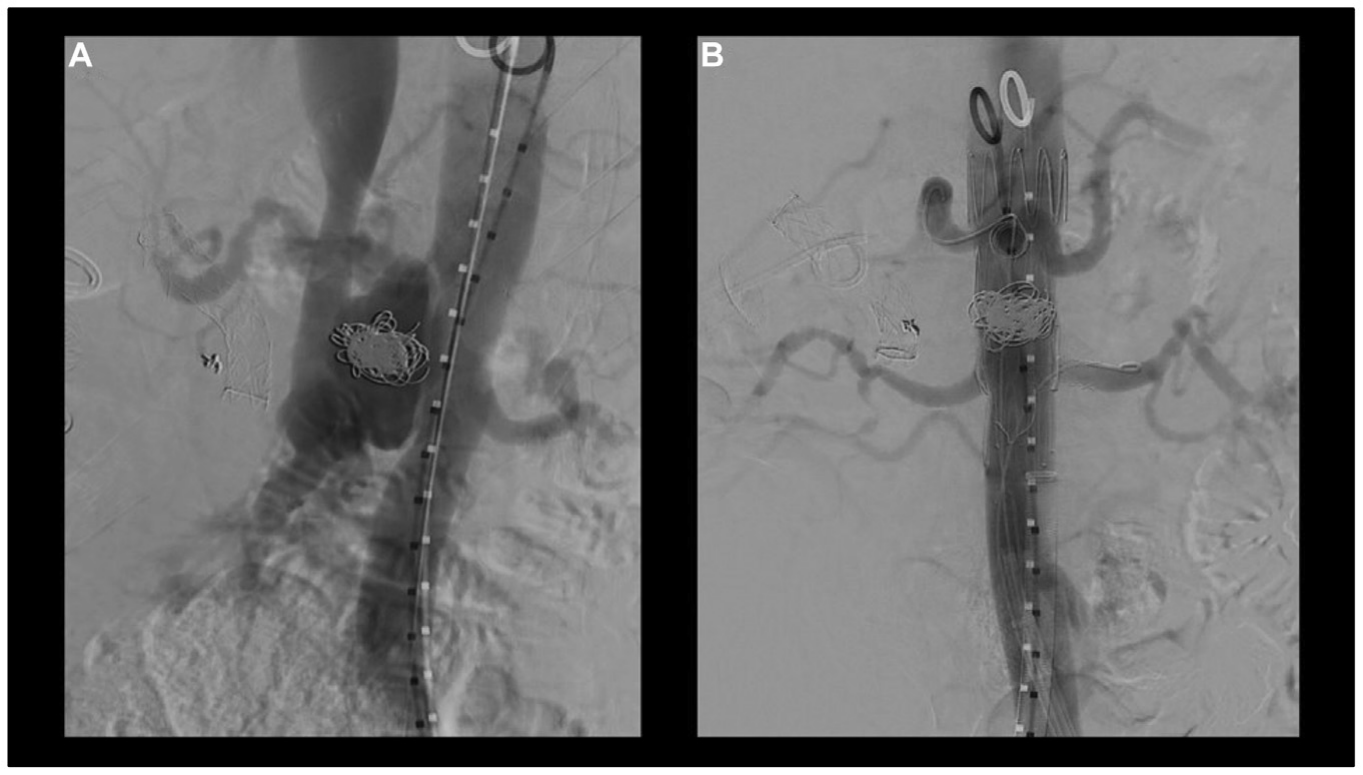
Before **(A)** and after **(B)**. Before **(A)** with pseudoaneurysm and arteriovenous fistula. After **(B)** demonstrating exclusion of pseudoaneurysm and resolution of fistula with flow through celiac and bilateral renal arteries. There is no evidence of endoleak.

A contrast angiogram was obtained above all stents at the level of the descending thoracic aorta distal to the left subclavian, revealing excellent flow into all visceral vessels and the distal aortoiliac section with resolution of the SMAVF fistula (Fig 3).

The patient recovered on the general ward. A CTA on postoperative day 2 revealed proper placement of the stent graft with resolution of the SMAVF. On the day of discharge (postoperative day 3), the patient was ambulating without assistance, voiding independently, tolerating a regular diet, and, of note, had nearly complete resolution of his bilateral leg edema. The patient was discharged with amoxicillin/clavulanic acid (Augmentin) ×1 month and aspirin 81 mg. Given the lack of systemic signs of infection and negative infectious workup (no elevated C-reactive protein, erythrocyte sedimentation rate, white blood cells, fevers, or positive blood cultures), lifetime antibiotics were not deemed appropriate.

At his 1-month follow-up, the patient was doing extremely well. He denied any abdominal pain or leg swelling. Interval CTA noted patent visceral stents with exclusion of the SMAVF as well as resolution of the peripancreatic collection (previously identified as blood based on the Hounsfield units).

## Discussion

Visceral arterial aneurysms and visceral arterial pseudoaneurysms (VAPAs) are rare yet potentially fatal pathologies with an estimated incidence between 0.01% and 0.2%.^1^^,^^2^ Among VAPAs, pseudoaneurysms of the SMA are even more uncommon, accounting for between 4.0% and 5.5% of all VAPAs.^2^^,^^3^ The most common etiology of VAPA formation is pancreatitis, likely resulting from leakage of proteolytic enzymes, which damage the vessel wall.^4^ Blunt, penetrating, or iatrogenic trauma from surgical procedures in close proximity to visceral vessels are also known causes of VAPA formation.^5^

Owing to the high rates of rupture associated with VAPA (estimated at 76.3% in one study),^6^ immediate intervention is required for the treatment of VAPA. Treatment options include open surgical repair, transcatheter selective embolization, covered stenting of the visceral artery, or thrombin injection. Transcatheter selective embolization has become the most common technique used in the treatment of VAPA and is associated with low rates of perioperative morbidity and mortality.^3^

In this report, we present the case of a patient who represented with an enlarging, symptomatic SMA pseudoaneurysm after coil embolization, after likely inadequate treatment of the initial pseudoaneurysm. Unique features of this case include the presence of a SMAVF and patient representation after coil embolization of the SMA.

SMAVF are exceedingly rare and are usually the result of blunt, penetrating, or iatrogenic trauma.^7^ Complications from untreated SMAVF include portal hypertension, high cardiac output heart failure, or bowel ischemia.^8^ In the present case, the patient did not present with any of the classic symptoms of SMAVF, likely owing to his prior history of SMA thrombosis and ligation, as well as the presence of visceral collaterals. He instead presented with lower extremity pain and swelling, likely owing to venous hypertension secondary to arterialization of the inferior vena cava.

Of the few case reports that exist on the repair of SMAVF in the setting of SMA pseudoaneurysm, treatment with coil embolization or covered stents was the preferred method of repair,^9^, ^10^, ^11^ with one report of open repair.^12^ To the best of our knowledge, this case is the first reported of SMA pseudoaneurysm with SMAVF treated with PMEG. The technical aspects of this case that contributed to successful repair include the use of a main body stent graft to occlude inflow to the SMA and encompass the celiac and renal vessels without impinging on the IMA.

## Conclusions

SMA pseudoaneurysm with SMAVF is an uncommon pathology associated with high rates of rupture and mortality. In this case, we illustrated the novel use of PMEG with exclusion of the SMA as a method to repair SMA pseudoaneurysm with SMAVF after prior thrombosis, ligation, and coil embolization of the SMA. For repair of SMA pseudoaneurysm with SMAVF, we recommend an individualized treatment approach tailored to each patient's particular anatomy and history, and offer PMEG as an option for endovascular repair when coil embolization or percutaneous stenting options fail.
